# Evolutionary mismatch along salinity gradients in a Neotropical water strider

**DOI:** 10.1002/ece3.7405

**Published:** 2021-04-09

**Authors:** Anakena M. Castillo, Luis F. De León

**Affiliations:** ^1^ Centro de Biodiversidad y Descubrimiento de Drogas Instituto de Investigaciones Científicas y Servicios de Alta Tecnología (INDICASAT‐AIP) Panamá Panamá; ^2^ Department of Biotechnology Acharya Nagarjuna University Guntur India; ^3^ Department of Biology University of Massachusetts Boston Boston MA USA

**Keywords:** evolutionary mismatch, fitness, local adaptation, maladaptation, osmotic niche, preadaptation, salinization

## Abstract

The evolution of local adaptation is crucial for the in situ persistence of populations in changing environments. However, selection along broad environmental gradients could render local adaptation difficult, and might even result in maladaptation. We address this issue by quantifying fitness trade‐offs (via common garden experiments) along a salinity gradient in two populations of the Neotropical water strider *Telmatometra withei*—a species found in both fresh (FW) and brackish (BW) water environments across Panama. We found evidence for local adaptation in the FW population in its home FW environment. However, the BW population showed only partial adaptation to the BW environment, with a high magnitude of maladaptation along naturally occurring salinity gradients. Indeed, its overall fitness was ~60% lower than that of the ancestral FW population in its home environment, highlighting the role of phenotypic plasticity, rather than local adaptation, in high salinity environments. This suggests that populations seemingly persisting in high salinity environments might in fact be maladapted, following drastic changes in salinity. Thus, variable selection imposed by salinization could result in evolutionary mismatch, where the fitness of a population is displaced from its optimal environment. Understanding the fitness consequences of persisting in fluctuating salinity environments is crucial to predict the persistence of populations facing increasing salinization. It will also help develop evolutionarily informed management strategies in the context of global change.

## Graphical abstract

Using common garden experiments, we quantify fitness trade‐offs along a salinity gradient in two populations of the Neotropical water strider *Telmatometra withei*—a species found in both fresh and brackish water environments across Panama. We found that populations seemingly persisting in high salinity environments might, in fact, be maladapted, following drastic changes in salinity. We suggest that variable selection imposed by salinization could result in evolutionary mismatch, where the fitness of a population is displaced from its optimal environment.

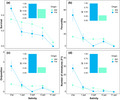

## 1 INTRODUCTION

The in situ persistence of populations in a changing environment depends largely on their ability to adapt to novel environmental conditions. Local adaptation occurs when selection favors the nonrandom association between a population's reproductive success (i.e., fitness) and the features of the environments that maximize that reproductive success (Anderson et al., 2013; Endler, 1986; Hereford, 2009; Kawecki & Ebert, 2004; Savolainen et al., 2007; Schluter, 2000), and it could occur through a combination of plasticity and genetic evolution (Anderson et al., 2013; Ashander et al., 2016; Burggren, 2018; Fournier‐Level et al., 2011; Jones et al., 2012; Lowry, 2012). Consequently, locally adapted populations tend to exhibit higher survival and fitness in their own “home” environment than in a “foreign” environment and vice versa (Hereford, 2009; Kawecki & Ebert, 2004). This prediction has been tested repeatedly via reciprocal transplant and common garden experiments (Gomez‐Mestre & Tejedo, 2003; Hereford, 2009) in both natural (Gomez‐Mestre & Tejedo, 2003; Leimu & Fischer, 2008; Savolainen et al., 2013) and human‐altered environments (Rolshausen et al., 2015).

However, making inferences about local adaptation in the context of broad environmental gradients remains challenging. First, classical studies of local adaptation tend to focus on populations that show (prior to experiments) trait divergence between alternative environments, where local adaptation is most likely to occur (Hereford, 2009; Schluter, 2000). Yet, a priori trait divergence is generally unknown for nonmodel species persisting along broad environmental gradients. Second, the contexts in which local adaptation is most often estimated represent highly divergent—yet binary—environmental gradients (Hereford, 2009) that impose stable (and perhaps predictable) selection pressures. Examples include low‐ and high‐predation sites (Endler, 1980, 1991; Reznick & Endler, 1982), benthic and limnetic zones of lakes (McPhail, 1993; Schluter & McPhail, 1992), or high and low salinity environments (Defaveri & Merila, 2014; Kozak et al., 2013; Wrange et al., 2014). By contrast, most natural environmental gradients are likely to be broad and highly variable, resulting in variable (and perhaps unpredictable) selection pressures. Thus, selection imposed by variable conditions could hinder local adaptation along broad environmental gradients, but this expectation remains understudied.

Salinity gradients provide a good model to test for the adaptive consequences of variable conditions on natural populations. Salinity levels experienced by freshwater organisms can vary anywhere from nearly fresh (<0.5 ppt) to brackish (0.5–30 ppt) and saline (30–50 ppt) water, and the exposure to these salinity levels can vary temporally, from hours to years (Gomez‐Mestre & Tejedo, 2003; Kozak et al., 2013). Previous studies have found evidence for local adaptation to high salinity levels in plants (Al‐Gharaibeh et al., 2017; Busoms et al., 2015), fishes (Defaveri & Merila, 2014; Kozak et al., 2013), and amphibians (Gomez‐Mestre & Tejedo, 2003), but most of these studies have been limited to narrow salinity gradients—generally comparing fresh versus brackish water populations, and only a few compare multiple salinity levels (Defaveri & Merila, 2014; Kozak et al., 2013). Furthermore, studies on the consequences of salinization for freshwater organisms have been limited to coarse taxonomic levels (i.e., above genus level), and to geographic regions historically affected by salinization (reviewed in (Castillo et al., 2017)). In fact, there are virtually no studies on the effect of salinization in Neotropical regions (Castillo et al., 2017), which contains a large portion of the planet's freshwater biodiversity (Abell et al., 2008).

The fluctuating nature of salinization could render local adaptation difficult if salinity changes overcome the adaptive potential of populations. That is, if populations lack phenotypic or genetic variation to cope with current changes in salinity, they are likely to undergo local extinction (Lewontin, 1974; Sinervo et al., 2010). In addition, even if populations manage to persist in newly salinized environments, their local fitness might be lower than expected in the ancestral freshwater environment, effectively rendering populations maladapted (Brady, 2013; Crespi, 2000; DeWitt & Yoshimura, 1998) to saline environments. In this case, maladaptation could be relative (Brady et al., 2019; Geladi et al., 2019; Hendry & Gonzalez, 2008; Hendry & Taylor, 2004; Rolshausen et al., 2015) or “partial,” in the sense that populations are able to persist, albeit with suboptimal fitness. This contrasts with absolute maladaptation (Geladi et al., 2019; Hendry & Gonzalez, 2008), where populations are unable to persist. Consequently, selection pressures imposed by fluctuating salinization could result in an “evolutionary mismatch” whereby the fitness of a population is displaced from its optimal environment (Hale et al., 2016; Lloyd et al., 2011; Negrin et al., 2019; Robertson et al., 2013; Schlaepfer et al., 2002). Understanding the persistence of populations along broad and variable environmental gradients requires a better understanding of the magnitude of adaptation and maladaptation along those gradients. Here, we use a combination of field surveys and common garden experiments to examine fitness trade‐offs along a salinity gradient in the Neotropical water strider *Telmatometra withei* in Panama.

## 2 MATERIALS AND METHODS

### 2.1 Study organism

**FIGURE 1:**
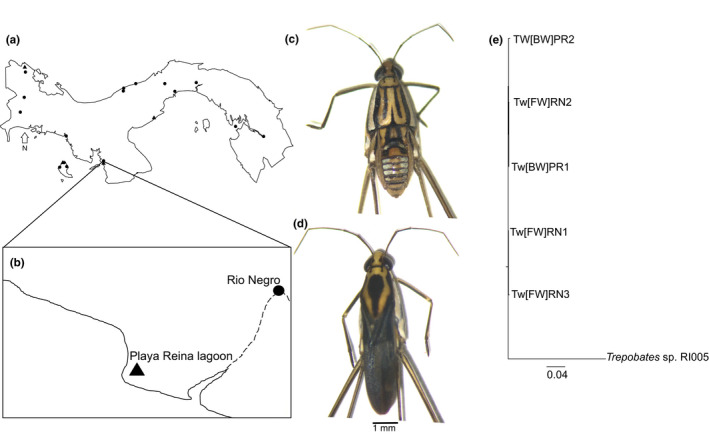
Sampling sites and geographic distribution of *Telmatometra withei* in Panama. Symbols represent fresh (circle) and brackish (triangle) water populations (a). Expanded area shows experimental sites (b). Two adult individuals are also shown in Panel c (wingless individual) and d (winged individual). Randomized Axelerated Maximum Likelihood phylogenetic tree based on COI gene (e)

*Telmatometra withei* (Bergroth, 1908) is a common water strider distributed from Ecuador to México (Molano et al., 2017; Pacheco, 2012; Padilla‐Gil, 2012), including the islands such as Puerto Rico and Trinidad and Tobago (Molano et al., 2017). Although *T. withei* is considered a freshwater species (Pacheco, 2012; Padilla‐Gil, 2012), we have found several populations inhabiting in a broad range of salinities, ranging from fresh to brackish water along the two slopes of the Isthmus of Panama, as well as on Coiba Island (Figure 1a). Our preliminary molecular analyses based on Mitochondrial COI found low genetic variation among populations (Figure 1e), which is consistent with the presence of a single species across salinity gradients in Panama. While some genera of saline‐adapted water striders are known (e.g., Genus *Halobates*) (Cheng, 2005; Harada, 2005), the potential for adaptation in typically freshwater species remains unexplored. For example, the Japanese water strider, *Aquarius paludum* (Kishi et al., 2006, 2009), and *Gerris thoracicus* from Finland (Kaitala, 1987; Vepsäläinen, 1978) are sometimes found in brackish waters, but their degree of local adaptation to high salinity environments has not been tested.

### 2.2 Study sites and experimental setting

Individuals of *T. withei* were collected from two sites located in Llano de Catival on the Western Azuero Peninsula on the Pacific coast of Panama (Figure 1a). The first site (Rio Negro [RN]; 7°38′22.0″N, 80°58′36.6″O) is a freshwater (FW) site, with gravel substrate, and is surrounded by secondary forest. The second site (Playa Reina lagoon [PR]; 7°37′31.1″N, 81°00′16.7″O) is a typical brackish water (BW) lagoon (~2 ppt), with sandy substrate, and is surrounded by mangrove forest and cativo (*Prioria* sp.) trees (Castillo et al., 2020). This site is influenced by both seawater intrusion (due to daily tidal fluctuations) and precipitation (during the rainy season), resulting in salinity levels that can range from 0.4 to 11 ppt (Figure 1b). At both sites, we collected adult individuals using a standard D hand net (mesh size: 500 μm) during the months of January to June of 2017 and 2018. Individuals were transported to the laboratory where they were acclimated (using water from their site of origin) for 24 hr before being transferred to experimental boxes. Experimental boxes were 12L (42.5 × 30.2 × 17.8 cm) for tolerance experiments, and 53L (58.4 × 41.3 × 31.4 cm) for common garden experiments. Each box was supplied with an air pump to promote oxygen circulation as well as a foam platform to facilitate resting and oviposition. Boxes were covered with a fine mesh to prevent water striders from escaping. For both experiments, we used natural filtered water from each study site as well as filtered seawater to prepare additional salinity concentrations. Filtering was performed using 500 µm mesh‐size sieve, which would remove most zooplankton and floating particles from the water. However, water striders were fed with *Drosophila* adults and eggs. At each study site, we used a YSI Pro Plus Multiparameter (YSI) to quantify standard physicochemical parameters, including temperature, conductivity, SPC (specific conductivity), total dissolve oxygen (TDS), pH, and salinity. These water parameters were also recorded in each experimental box weekly (Table 1 and 2).

**TABLE 1:** Environmental parameters (Range, Mean ± *SE*) at each sampling site

Environmental parameters	Sites				
	Rio Negro		Playa Reina lagoon		*p*‐value
	Range	Mean ± *SE*	Range	Mean ± *SE*	
Temperature (T°)	24.2–28.7	26.44 ± 0.6	25.4–29.6	27.9 ± 0.8	.16
Specific conductivity (uS/cm)	107–234	122.9 ± 11.9	321–17002	2,952.6 ± 2,348.5	.34
Total dissolved solids (mg/L)	63–123	77.4 ± 9.3	162–12320	2035.6 ± 1716.2	.36
pH	8.2–8.7	8.6 ± 0.1	7.7–8.6	8.1 ± 0.1	.11
Salinity (ppt)	0.05–0.08	0.07 ± 0.01	0.22–11	1.90 ± 1.50	.52

**TABLE 2:** Environmental parameters in experimental treatments for freshwater and brackish populations

Treatment	T° (i–f)	SPC (i–f)	TDS (i–f)	pH (i–f)	ppt (i–f)
Freshwater population					
FW	25.5–25.9	69–102	44.1–65.3	8.3–8.4	0.04–0.05
1 ppt	25.6–25.9	1997–2140	1280–1369.6	7.8–7.9	1.15–1.23
3 ppt	24.6–25.7	3,583.8–5690	2290–3640	8.2–8.0	3.04–3.14
5 ppt	25.3–25.7	7,287.4–9320	4660–5964.5	7.9–8.0	5.0–5.3
11 ppt	28.9–28.0	18601–19500	11903–12503	8.4–8.3	11.02–11.07
Brackish population					
FW	29.2–25.9	80.0–86	123.0–128.0	9.1–8.5	0.03–0.04
1 ppt	25.0–25.4	1848.2–1742	1,202.4–1577.3	7.9–8.3	1.01–1.31
3 ppt	22.7–22.9	2,648.9–4865	2006.8–3765.7	7.0–8.2	2.94–3.20
5 ppt	26.2–25.9	7,561.7–7738	4840–4950	7.7–7.8	5.85–6.36
11 ppt	26.0–24.9	19612–20430	12727–13305.5	7.9–7.8	11.05–12.01

Shown are initial (i) and final (f) values for T° (temperature in °C), SPC (specific conductivity), TDS (total dissolve oxygen), pH, and ppt (salinity).

### 2.3 Morphological identification and DNA barcoding

Adult specimens were identified using a standard taxonomic key (Molano et al., 2017; Pacheco, 2012); (Figure 1c,d). Additionally, to confirm species identification as well as to explore genetic variation across populations, we amplified the standard COI barcoding fragment in 2–3 individuals from each population (Figure 1e). Total DNA was extracted from the full body of each individual using the standard Qiagen DNeasy Blood and Tissue kit. The barcoding fragment (COI) was amplified using the following pairs of primers: LCO (1490) and HCO (2198); dg LCO (1490) and dg HCO (2198) (Ebong et al., 2016). Multiple alignments were made using the ClustalW algorithm, according to the default settings (Ebong et al., 2016). We then ran a Randomized Axeelerated Maximum Likelihood analysis, using the nucleotide model GTR+G+I, with 1,000 bootstrap replicates and parsimony random seed set to 1 (Ebong et al., 2016). Finally, a phylogenetic tree was built using *Trepobates* sp. as outgroup in Geneious version 10.0.6. The sequence of *Trepobates* sp. was obtained from De León et al. (2020) (GenBank accession number: KX039636.1).

### 2.4 Salinity tolerance experiments

To examine salinity tolerance in both fresh and brackish water populations, we estimated LC_50_ values over a period of 48, 72, and 96 hr. LC_50_ represents the salinity concentration at which 50% of the sampled population exhibit mortality (Sparks, 2000). For these experiments, we estimated LC_50_ for the following salinity concentrations: 0 (freshwater), 1, 3, 5, 10, 15, 20, 25, and 33 (seawater) ppt. Each salinity treatment was replicated five times, with each replicate containing a total of 10 adult individuals. Visual censuses were performed at 48, 72, and 96 hr, and LC_50_ values were estimated based on the number of individuals that survived at each time interval, following (Gomez‐Mestre & Tejedo, 2003; Kefford et al., 2004). Dead individuals were removed from the experimental boxes to maintain water quality. These experiments had two main goals: (a) determining the maximum salinity tolerance of both fresh and brackish water populations of *T*. *withei*, and (b) using this tolerance (i.e., realized LC_50_ values) as a threshold for our common garden experiments.

### 2.5 Common garden experiments

To estimate fitness trade‐offs along a salinity gradient, we performed standard common garden experiments (Gomez‐Mestre & Tejedo, 2003). Ten adult individuals (five females and five males) from both FW and BW populations were transplanted to experimental boxes with the following salinity concentrations: 0, 1, 3, 5, and 11 ppt. We did not perform experiments beyond 11 ppt because our pilot study found virtually no survival at those salinity concentrations (see section 3). Experimental boxes for 0 ppt were prepared with filtered water from Rio Negro, the “home” site of the FW population. Experimental boxes at 1 ppt were prepared with filtered water from Playa Reina lagoon, the “home” site for our BW population. The remaining salinity concentrations (3, 5, and 11 ppt) were prepared by combining filtered seawater and freshwater from Rio Negro. For each salinity treatment, we performed 8–10 replicates for the FW population and 8 replicates for BW population. During the first 30 days of the experiments, we monitored the following fitness (*W*) surrogates daily: adult survival (estimated as the ratio between the number of survival individuals and the initial number of individuals), fecundity (number of eggs), oviposition rate (number of eggs per day), and number of offspring (representing the number of F1 juveniles, before wing development). After this period, surviving adults were removed from the experimental boxes, but we continued to monitor offspring survival (F1) to maturity to get an estimate of longevity until 90 days. We also estimated egg size for a subset of the eggs from the FW (*n* = 27) and BW (1 ppt; *n* = 30) populations, using digital photographs and ImageJ v1.51 (Rasband, 1997–2012).

### 2.6 Magnitude of local adaptation and maladaptation

We used all fitness‐related traits (survival, fecundity, oviposition rate, and number of offspring [F1]) from common garden experiments to quantify local adaptation for both FW and BW populations in each of their home environments with the following equation from (Hereford, 2009).

(1) $$\text{LA}=\frac{\left(W_{\text{native population}\;}‐W_{\text{foreign population}\;}\right)}{\text{avg}\left(W_{\text{native site}}\right)}$$

where *W* represents the mean fitness of the native and the foreign population at the native population's site, and avg (*W*) represents the mean fitness across both populations at that site (Hereford, 2009). Positive and negative values indicate local adaptation and maladaptation for the focal native populations, respectively (Hereford, 2009).

As a complementary approach, we then inferred the magnitude of maladaptation by estimating the proportional fitness difference between the ancestral freshwater population and the derived brackish population. To quantify this parameter, we used the following formula:

(2) $$\text{MA}=W_{\text{ideal}}‐W_{\text{realized}}$$

representing the difference between the mean fitness of the ancestral (reference) population in its home environment standardized to 1.0 (*W*_ideal_; here, the freshwater population) and the fitness of the derived population in its home environments (*W*_realized_; here, the brackish water population), with MA between 0 and 1 indicating 0% and 100% maladaptation, respectively. These estimates assume that the ancestral population experiences an optimal fitness in its home environment, which is a simplified assumption, given that the environment may change constantly, and thus, populations might not always be near the optimum. In addition, even if the derived population shows lower fitness values in the novel environment, this difference may still be adaptive. However, comparing the proportional fitness difference between both populations under similar experimental condition will give an indication of the magnitude of fitness loss in the derived population in the novel environment.

### 2.7 Data analysis

To estimate salinity tolerance for both fresh and brackish water populations, we performed logistic regressions between survival and salinity. Survival was estimated as the ratio between the number of survival individuals and the initial number of individuals in each experiment, and LC_50_ thresholds were obtained from the regression equation. We estimated LC_50_ independently for each FW and BW population, as well as for each time interval (48, 72, and 96 hr). We then ran ANCOVAs to test for variation in salinity tolerance (here LC_50_) as a function of population of origin, salinity level, and their interaction. We also estimated the proportion of variance (*R* ^2^) explained by each of the models.

To test for variation in individual fitness surrogates (fecundity, oviposition rate, and number of offspring [F1]) as a function of salinity levels (FW, 1, 3, and 5 ppt) in the common garden experiments, we performed analyses of variance (ANOVAs), followed by Tukey's HSD Post hoc tests for each trait independently. To explore local adaptation in both FW and BW populations, we performed (for each trait) Generalized Linear Mixed Effect Models (GLMEMs), with site, population, and sex included as fixed factors, and box number as random factor. With these models, we tested for variation in fitness surrogates as a function of population of origin (population effect), treatments (site effect), and their interaction (local adaptation). Survival data were analyzed using logistic regression. Finally, we performed Kaplan–Meier analyses to quantify the temporal pattern of survival (in days) of at least 50% of individuals from both FW and BW environments across salinity treatments. All analyses were performed in R Development Core, 2008.

## 3 RESULTS

### 3.1 Salinity tolerance experiments

**FIGURE 2:**
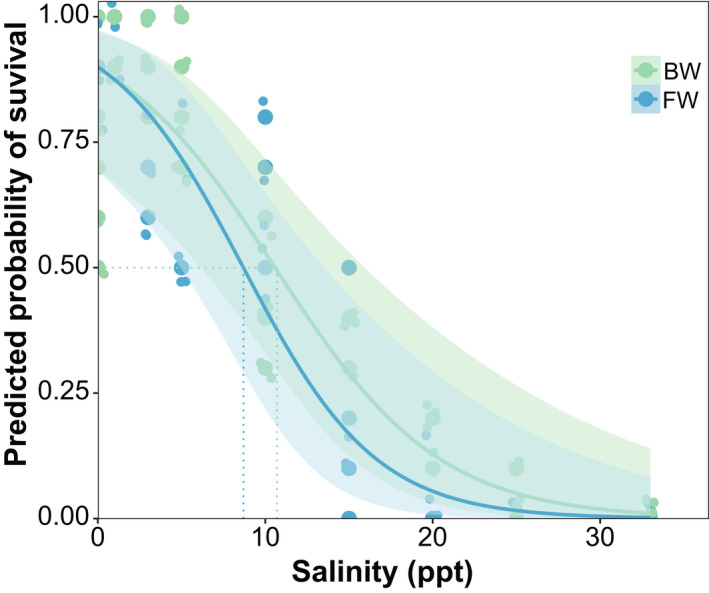
Experimental estimates of salinity tolerance in *Telmatometra withei*. The curves represent mortality of freshwater (green line) and brackish (blue line) populations along a salinity gradient during 48‐hr experiments. Points represent experimental boxes, and the dotted lines indicate LC_50_ values for both populations. Shaded area represents 95% confidence intervals

Salinity had a significant effect on survival of *T. withei*, with both FW and BW populations reaching 50% mortality (LC_50_ ~48 hr) at relatively low salinity levels (Table 3). Interestingly, LC_50_ tended to be lower for the FW (8.69 ppt) than BW (10.58 ppt) populations, although this difference was not statistically significant (Table 3; Figure 2; *F*_1,86_ = 1.65; *p* = .20). This pattern of mortality was sustained after 72 and 96 hr of exposure, with LC_50_ values decreasing to ~ 5 and 6 ppt for FW and BW populations, respectively (Table 3). Our ANCOVA also showed a significant effect of salinity on LC_50_, but there was no effect of population of origin or their interaction (Table 3).

**TABLE 3:** Salinity tolerance in *Telmatometra withei*

Population	Exposure time					
	48 hr		72 hr		96 hr	
	LC_50_	*R* ^2^	LC_50_	*R* ^2^	LC_50_	*R* ^2^
Freshwater	8.69 ± 0.57	80.5	6.06 ± 0.50	75.6	4.73 ± 0.45	66.6
Brackish water	10.58 ± 0.66	83.5	6.44 ± 0.49	70.0	5.77 ± 0.45	66.9

Source	*F*	*p*	*F*	*p*	*F*	*p*
Population	1.65	.20	0.09	.75	0.65	.42
Salinity	391.37	**<.001**	228.81	**<.001**	172.25	**<.001**
Pop × Salinity	0.05	.82	0.08	.77	0.43	.51

Note

The top table shows mean and standard error of LC_50_ values (ppt ± *SE*) per population at 48, 72, and 96 hr of exposure to different salinity levels. The lower table shows variation in LC_50_ across population of origin, salinity levels, and their interaction based on ANCOVA. *R* ^2^ represents the fit of the model, and bold indicates statistical significance

### 3.2 Common garden experiments

**FIGURE 3:**
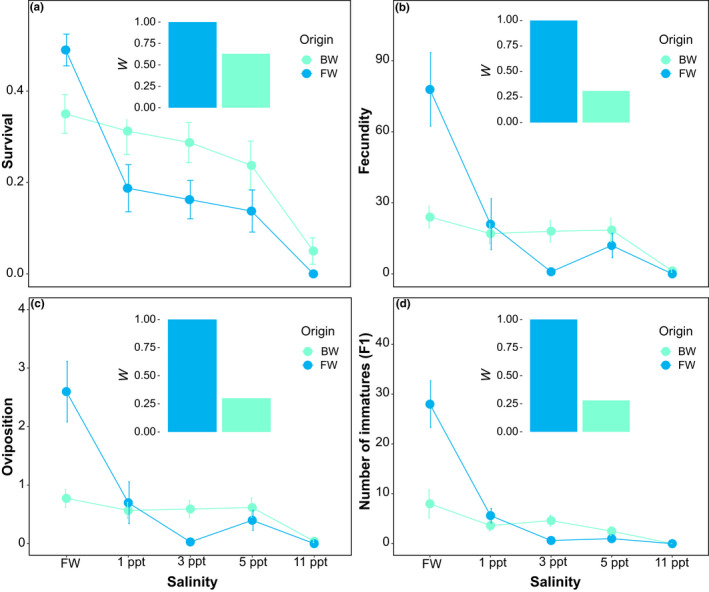
Experimental estimates of fitness trade‐offs along a salinity gradient in the water strider *Telmatometra withei*. Several fitness correlates are shown: survival (panel a), fecundity (panel b), oviposition rate (panel c), and number of immatures (panel d). Error bars show mean and standard error. Inner plots show the ideal fitness (*W*_ideal_) of the freshwater population (i.e., the ancestral FW population in its home FW environment) and the realized fitness (*W*_realized_) of the brackish water populations (i.e., the derived BW population in its home BW environment), with the difference between the two values representing the degree of maladaptation for the brackish water population (see section 2)

**FIGURE 4:**
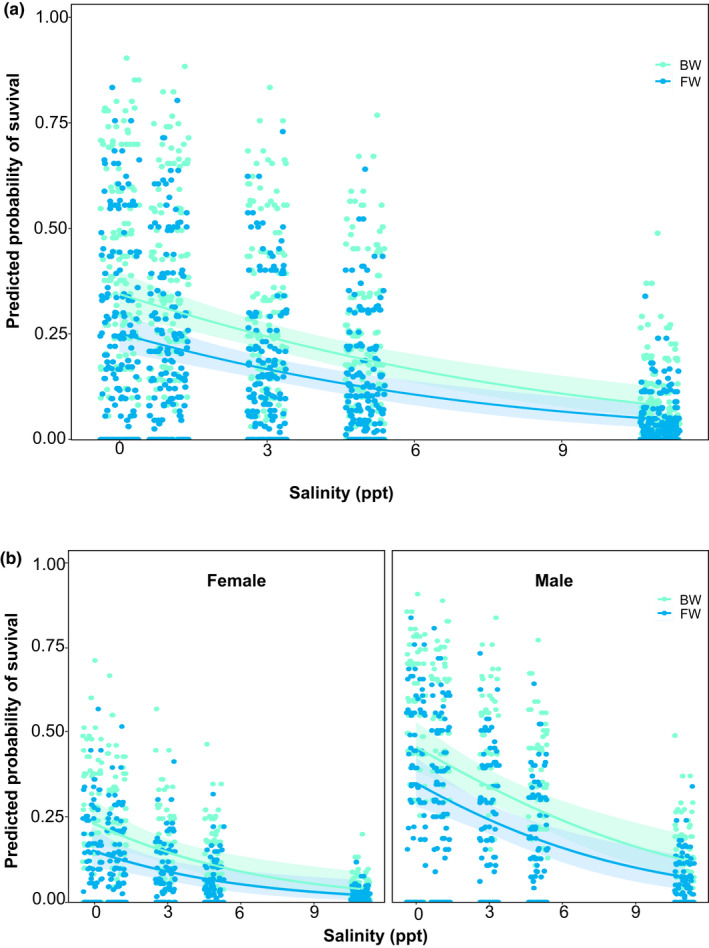
Probability of survival in *Telmatometra withei* along a salinity gradient based on common garden experiments. Panels show logistic regressions across the entire data set (a), and by sex (b). Shaded area represents 95% confidence intervals

Salinity had a significant effect on fitness correlates (Figure 3; Table 4), but this effect varied between populations, sex, and across salinity levels. Specifically, the four traits (survival, fecundity, oviposition rate, and the number of offspring [F1]) showed statistically significant declines (~80%) in the FW population raised in the foreign BW environment, but not in the BW population across any of the environments. The only exception to this pattern was the 11 ppt treatment in which fitness decreased to nearly 0% for both populations, although survival in the BW population was ~5% (Figure 3; Table 4). When comparing both populations across salinity levels, we found higher fitness overall in the FW population in its home FW environment than either the FW or BW population across any of the salinity treatments. However, the BW population tended to show higher fitness than the FW population in high salinity treatments (1–11 ppt), and this difference was consistently significant at 3 ppt (Figure 3; Table 4). A similar pattern was observed by sex, with both males and females from the FW population showing overall higher survival in their home environment, and the BW population showing higher survival (for both males and females) at higher salinities (3 and 5 ppt). Interestingly, only males from BW population tended to survive at 11 ppt (Figure 4; Figure S1a,b).

**TABLE 4:** Variation in fitness correlates along a salinity gradient in *Telmatometra withe*

Traits	FW			1 ppt			3 ppt				5 ppt		
	*N*	M	±*SE*	*N*	M	±*SE*	*N*		M	±*SE*	*N*	M	±*SE*
Freshwater population													
Survival (overall)	10	0.49^a^	0.03	8	0.19^b^	0.05	8		0.16^b^	0.04	8	0.14^b^	0.05
Male	10	0.33^a^	0.03	8	0.14^b^	0.04	8		0.15^b^	0.04	8	0.11^b^	0.04
Female	10	0.16^a^	0.04	8	0.05^b^	0.02	8		0.01^b^	0.01	8	0.03^b^	0.02
Fecundity	10	77.90^a^	15.54	8	21.00^b^	10.75	8		0.88^b^	0.40	8	12.00^b^	5.14
Oviposition rate	10	2.60^a^	0.52	8	0.70^b^	0.36	8		0.03^b^	0.01	8	0.40^b^	0.17
Number of immatures	10	28.00^a^	6.23	8	5.63^b^	1.45	8		0.63^b^	0.32	8	1.00^b^	0.57
Brackish population													
Survival (overall)	8	0.35^a^	0.04	8	0.31^a^	0.15	8	0.29^a^		0.04	8	0.24^a^	0.05
Male	8	0.24^a^	0.05	8	0.20^a^	0.03	8	0.25^a^		0.04	8	0.13^a^	0.05
Female	8	0.11^a^	0.04	8	0.11^a^	0.04	8	0.04^a^		0.01	8	0.11^a^	0.05
Fecundity	8	24.00^a^	4.50	8	17.00^a^	4.13	8	18.00^a^		4.54	8	18.50^a^	5.02
Oviposition rate	8	0.77^a^	0.15	8	0.57^a^	0.14	8	0.60^a^		0.14	8	0.61^a^	0.17
Number of immatures	8	8.00^a^	2.83	8	3.63^a^	1.05	8	4.63^a^		1.13	8	2.50^a^	0.87

Note

The data represent mean (M) and standard error (±*SE*) for different fitness correlates at different salinity levels. Letters denote significant differences at *p* < .05 based on ANOVAs, followed by Tukey's HSD tests.

The number of immatures in the BW population showed a twofold increase when they were raised in the foreign FW environment, although this increase was not as high as that of the FW population in the same environment (Figure 3d; Table 4). In addition, there were no statistical differences in egg size between both populations (*t*_(51)_ = −1.73, *p* = .08; Figure S2). Overall, these results were confirmed by our GLMEMs, which showed significant differences in the four fitness correlates across treatments, as well as an interaction between treatment and population of origin. In addition, the number of immatures showed significant differences between populations of origin, and survival showed a significant effect of sex (Table 5).

**TABLE 5:** Salinity effect on fitness correlates in *Telmatometra withei*

Variables	Sum Sq	*F*	*χ* ^2^	Pr (χ^2^)
Survival				
Treatment	1.39	24.31	0.35	**<.001**
Sex	0.39	41.05	0.39	**<.001**
Origin	0.04	3.07	0.04	.08
Treatment: origin	0.22	3.73	0.05	**.01**
Fecundity				
Treatment	28,234	14.38	7,058	**<.001**
Origin	1,285	2.62	1,285	.11
Treatment: origin	13,038	6.64	3,259	**<.001**
Oviposition rate				
Treatment	30.99	14.22	7.75	**<.001**
Origin	1.51	1.51	2.77	.10
Treatment: origin	14.79	3.70	6.79	**<.001**
Number of immatures				
Treatment	4,099	24.15	1,024.70	**<.001**
Origin	291	7.41	290.60	**.01**
Treatment: origin	1576	10.05	394.10	**<.001**

Note

Values represent the results from individual Generalized Linear Mixed Effect Models (GLMEMs) on fitness‐related traits from common garden experiments. Statistical significance was evaluated via separated ANOVAs. Statistical significance is shown in bold.

These results were supported by our Kaplan–Meier analysis that showed that 50% of the individuals from the FW population were likely to survive for at least 30 days in their home FW environment, but only ~5–10 days at 1–5 ppt, and 2 days at 11 ppt (Figure S4a). By contrast, 50% of the individuals from the BW population were likely to survive for at least 20 days in their home BW environment, up to 18 days at other salinities (FW and 3–5 ppt), and ~4 days at 11 ppt (Figure S4b).

### 3.3 Local adaptation and maladaptation

**FIGURE 5:**
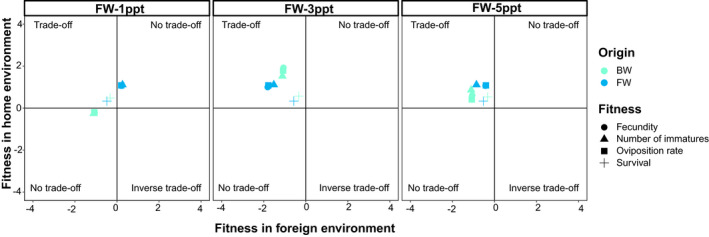
Patterns of local adaptation of fresh and brackish water populations of *Telmatometra withei* along a salinity gradient. Each panel shows the fitness advantage for the freshwater population (blue) in its home (FW) and foreign (1, 3, and 5 ppt) environment, and conversely, for the brackish water population (green) in its home (1, 3, and 5 ppt) and foreign (FW) environment cross fitness‐related traits: survival (cross), fecundity (circle), oviposition rate (square), and number of immatures (triangle). Populations show local adaptation to either home (upper left corner; trade‐off) or foreign (lower right; inverse trade‐off) environment only, to both (upper right corner; no trade off) or to neither environment (lower left corner; no trade‐off)

We found evidence for local adaptation (LA) in both FW and BW populations. For the FW population, we found strong LA in its home environment for survival (LA _FW_ = 0.33), fecundity (LA_FW (home)_ = 1.06), oviposition rate (LA_FW (home)_ = 1.08), and number of offspring (LA_FW (home)_ = 1.11). The FW population also showed weak LA at 1 ppt for fecundity (LA_FW in 1 ppt_ = 0.21), oviposition (LA_1 ppt_ = 0.20), and number of immatures (LA_1 ppt_ = 0.26), but not for survival (LA_FW in 1 ppt_ = −0.48). The FW population also showed evidence for maladaptation across traits at 3 ppt (LA_FW average_ = −1.42) and 5 ppt (LA_FW average_ = −0.56). In addition, we found evidence for fitness trade‐offs (LA to the home environment) between environments across traits at 3 and 5 ppt, as well as for survival at 1 ppt (Figure 5).

For BW population, we found weak LA in high salinity environments (3 and 5 ppt) for survival (LA_BW in 3 ppt_ = 0.57, LA_BW in 5 ppt_ = 0.53), fecundity (LA_BW in 3 ppt_ = 1.81, LA_BW in 5 ppt_ = 0.43), oviposition rate (LA_BWin 3 ppt_ = 1.78; LA_BW in 5 ppt_ = 0.41), and number of immatures (LA_BW in 3 ppt_ = 1.52, LA_BW in 5 ppt_ = 0.86). We also found evidence for maladaptation across traits in both the FW (LA_BW average_ = −0.90) and 1 ppt (LA_BW average_ = −0.22) treatments, except for survival at 1 ppt (LA_BW in 1 ppt_ = 0.48). In addition, we found evidence for fitness trade‐offs (LA to the home environment) across traits for 3 and 5 ppt (Figure 5).

Finally, we found a high magnitude of maladaptation in the BW population in its home BW environment across traits and salinities (MA_survival_ = 0.37, MA_fecundity_ = 0.69, MA_oviposition rate_ = 0.70, and MA_number of offspring (F1)_ = 0.72; Figure 3).

## 4 DISCUSSION

Salinization due to sea‐level rise is an increasing challenge for freshwater biodiversity. However, the extent to which freshwater organisms might be able to adapt to these changes is not well understood, particularly in Neotropical environments (Castillo et al., 2017). We explored this issue by quantifying fitness trade‐offs along a salinity gradient in two populations of the Neotropical water strider *T. withei*. We observed a strong effect of salinity on survival and reproductive traits for both FW and BW populations. The FW population showed strong fitness trade‐offs along salinity levels, with evidence for local adaptation to its home FW environment, but not to high salinity levels. The BW population also showed fitness trade‐offs along salinity levels, with evidence for weak local adaptation (for survival only) across salinity levels (1–5 ppt). However, the overall fitness of the BW population was only a fraction of that of the FW population in its home FW environment, indicating a high magnitude of maladaptation in the population persisting in BW environments. A similar pattern was observed when examining survival by sex, although males tended to show higher survival than females. In the following, we discuss the implication of these findings.

### 4.1 Salinity tolerance in *T. withei*

Although salinity is an important factor structuring aquatic biodiversity (Cañedo‐Argüelles Iglesias, 2020; Cañedo‐Argüelles et al., 2012, 2016, 2019; Castillo et al., 2017; Herbert et al., 2015; Hintz et al., 2017; Hintz & Relyea, 2019; Szöcs et al., 2014), salinity tolerance is most often studied at broad taxonomic scales (Castillo et al., 2017). Thus, the selective role of salinity (i.e., its fitness consequences) has been less explored in coastal freshwater organisms (Gomez‐Mestre & Tejedo, 2003; Kishi et al., 2006, 2009; Kozak et al., 2013). Here, we showed that salinity has a strong effect on survival of *T. withei*, with both FW and BW populations experiencing 50% mortality at salinities as low as 4 and 5 ppt, respectively. This is consistent with studies of temperate water striders (*A. paludum*, (Kishi et al., 2009; Kishi et al., 2006; Kishi et al., 2007); *Gerris latiabdominis*, (Kishi et al., 2013)), which are often found in similar salinity levels. This indicates that freshwater water striders are, in general, able to cope with some degree of salinization, with some species even inhabiting the open ocean (*Halobates*; (Cheng, 2005; Harada, 2005)).

However, given the fluctuating nature of salinization in coastal freshwaters, one remaining question is the extent to which salinity tolerance (here based on LC_50_ values) can help predict long‐term population persistence along broad salinity gradients, especially as sea levels rise. If so, salinity tolerance estimates could inform the extent of local adaptation in populations experiencing increased salinization. At our BW site (Playa Reina lagoon), salinity in the field ranged from ~1 to 11 ppt, indicating that BW populations are likely to experience a broad range of salinities. In fact, during our sampling, water striders were common at this site even when salinity was 11 ppt. However, LC_50_ estimates from our laboratory experiments were relatively low (~5 ppt), and we observed virtually no survival beyond 11 ppt. In addition, there were no significant differences in LC_50_ values between FW and BW populations. This suggests a degree of “mismatch” (i.e., environmental mismatch) between the osmotic tolerance of populations and the range of salinities they experience in natural environments.

This also suggests that salinity tolerance experiments are good indicators of the upper osmotic tolerance of populations (here 11 ppt), which can inform experimental settings to explore adaptation to saline environments. However, the short‐term nature of tolerance experiments and their focus on immediate survival rather than life‐long reproductive success is likely to underestimate the fitness consequences of salinization in typical freshwater organisms (see the following section).

### 4.2 Magnitude of adaptation and maladaptation

Selective pressures imposed by divergent environments often result in local adaptation, where populations evolve higher fitness in their own “home” environment than in the alternative “foreign” environment and vice versa (Endler, 1986; Hereford, 2009; Kawecki & Ebert, 2004; Schluter, 2000). However, the evolution of local adaptation along broad (and sometimes fluctuating) environmental gradients is likely more challenging (Gomez‐Mestre & Tejedo, 2003; Polechová et al., 2009). This is because fluctuating environments are likely to result in variable strength and direction of selection (Grant & Grant, 2002), which could overcome the adaptive potential of populations (Brady, 2013; DeWitt & Yoshimura, 1998; Fox & Harder, 2015; Sinervo et al., 2010), particularly if migration is not an option (Atkins & Travis, 2019; Kleynhans et al., 2016).

In addition, previous work suggests that in variable environments, plasticity is more likely to evolve than a fixed trait (Ashander et al., 2016; Burggren, 2018; Ghalambor et al., 2007; Hadfield, 2016; Via & Lande, 1985). In the case of salinization, populations might experience variable levels of salinity, ranging from fresh to highly saline waters (e.g., ~0.22–11 ppt at Playa Reina lagoon), which could result in periodic “mismatches” between the fitness of a population and its optimal osmotic niche (Gomez‐Mestre & Tejedo, 2003; Negrin et al., 2019). Thus, populations seemingly persisting in specific salinity levels might in fact be maladapted, following drastic changes in salinity.

We explored this issue by quantifying the magnitude of local adaptation (i.e., fitness trade‐offs along salinity levels; (Hereford, 2009)) as well as the “magnitude of maladaptation” (i.e., fitness differences between the ancestral FW population and the derived BW population in their home environments). Using these metrics, we found that the BW population showed apparent local adaptation to saline environments (1–5 ppt), but only for survival. However, its overall reproductive success was ~60% lower than that of the ancestral FW population in its home environment, suggesting a high magnitude of maladaptation in the BW population. Indeed, its overall life‐long fitness (based on the number of offspring) was significantly higher when it was raised in the FW treatment (Figure S3a), perhaps suggesting that the BW population is persisting away from the species' optimal osmotic niche. Thus, the physiological challenges imposed by osmoregulation in saline environments (Kozak et al., 2013; Potts & Parry, 1964; Rivera‐Ingraham & Lignot, 2017; Sutcliffe, 1961) are likely to constraint the evolution of local adaptation in those environments. This pattern is consistent with an evolutionary mismatch (Hale et al., 2016; Lloyd et al., 2011; Marshall et al., 2010; Negrin et al., 2019; Robertson et al., 2013; Schlaepfer et al., 2002), whereby drastic environmental disturbances might overcome the adaptive potential of populations (Polechová & Barton, 2015; Polechová et al., 2009). In the case of *T. withei*, adaptation to saline environments could be limited by potential trade‐offs between reproduction and survival. This was indicated by the fact that the BW population showed substantial survival in the high salinity treatments, but its overall fecundity and number of offspring were extremely low in the same treatments. Similarly, the temperate water strider *G. thoracicus* is known to show high longevity (a trait associated with survival), but low reproductive output in treatments with low food supply (Kaitala, 1987), suggesting that water striders can effectively trade‐off reproduction for survival when faced with stressful environments. This also suggests that our observation of high adult survival in saline environments (both in the field and in the common garden experiments) may reflect phenotypic plasticity, rather than local adaptation. However, more work is needed to confirm this possibility.

Another possibility is the existence of preadaptation of the BW population to the ancestral FW environments (Geladi et al., 2019). This could occur if the BW population is able to retain genetic variation associated with survival in the FW environments. In addition, given that the BW environment is highly variable, the BW population is likely to experience a broad range of salinities, including freshwater. At a broader scale, although freshwater salinization due to climate change is expected to increase globally (Courchamp et al., 2014; IPCC, 2007; IPPC, 2000), salinization could also decrease in areas with high precipitation (Gomez‐Mestre & Tejedo, 2003; Short et al., 2016; Wrange et al., 2014). Therefore, retaining ancestral polymorphism associated with FW environments (i.e., preadaptation) could facilitate persistence of populations in these fluctuating environments. However, preadaptation to ancestral environments could also be costly, and it could compromise the evolution of local adaptation in novel environments (Atkins & Travis, 2019). Another possibility is gene flow, which could constrain local adaptation in novel environments (Farkas et al., 2015; Hendry & Taylor, 2004; Hendry et al., 2002; Kawecki & Ebert, 2004). In this case, gene flow from the FW population could swamp adaptation to high salinity environments—a likely possibility in our system, given the proximity between populations and the downstream location of the BW population.

An important question is how can maladapted (or partially adapted) populations persist in the face of increased salinization? Maladaptation to a stressful environment could be overcome ex situ (Bolnick & Nosil, 2007; Farkas et al., 2016; Lenormand, 2002) if populations are able to disperse to less stressful environments (Defaveri & Merila, 2014; Farkas et al., 2015). This is certainly a possibility for *T. withei*, given that we have observed in the field a high frequency (11%) of winged individuals in the BW population (Figure 1d), in contrast to FW populations (<1.5%; Figure 1c). In other water strider species (*A. paludum*, (Kishi et al., 2007; Kishi et al., 2013)), wing development has also been associated with changes in salinity (Kishi et al., 2006, 2007, 2009), which could allow for dispersal to less saline environments (Kishi et al., 2006, 2007). Thus, perhaps a combination of partial adaptation and dispersal and recolonization is a likely mechanism promoting persistence of populations in these fluctuating environments. Another possibility is phenotypic plasticity rather than genetic adaptation. For instance, similar to other systems (Ashander et al., 2016; Burggren, 2018; Crispo et al., 2010), plasticity could facilitate persistence of populations along salinity gradients, which could buy time for adaptation to evolve, a possibility that requires further research.

### 4.3 Future work

Although we showed evidence for both adaptation and maladaptation in *T. withei*, we consider these results as preliminary, given that only two populations were included in our analyses. Thus, several questions remain to be explored. For instance, what are the physiological consequences of salinization as well as the plastic or genetic mechanism underlying local adaptation in *T. withei*. In addition, what is the extent of gene flow across FW and BW populations, and how it might promote or constraint adaptation (Farkas et al., 2015; Hendry & Taylor, 2004; Kawecki & Ebert, 2004) in this system is an open question. Finally, the role of demographic factors such as population size in mediating population persistence (Bell & Gonzalez, 2011; Gomulkiewicz & Holt, 1995) in *T. withei* needs to be considered.

Overall, although more work is clearly needed, our analysis of fitness trade‐offs along a salinity gradient revealed several aspects of local adaptation that are difficult to observe in studies of discrete environments. First, adaptation to extreme salinities in *T. withei* may be limited, given that both FW and BW populations failed to survive at salinities beyond 5 ppt. Thus, persistence of populations in high salinity environments may be facilitated by phenotypic plasticity rather than local adaptation. Second, if it occurs, local adaptation to broad and fluctuating environmental gradients is costly (Hereford, 2009), and could result in maladaptation to those environments. Third, preadaptation to ancestral environments is important in determining the magnitude of local adaptation in novel‐disturbed environments. Finally, dispersal ability could facilitate persistence of seemingly maladapted populations along variable environmental gradients.

## 5 CONCLUSION

In summary, our results based on two populations of the Neotropical water strider *T. withei* suggest that variable conditions along environmental gradients such as salinization of coastal freshwaters are likely to result in evolutionary mismatch, where the fitness of a population is periodically decoupled from its optimal environment. From a theoretical perspective, quantifying the magnitude of adaptation and maladaptation along environmental gradients will inform the role of adaptive evolution in the persistence of biodiversity in variable environments. From a practical perspective, it will allow the development of “evolutionary‐informed” management strategies to address biodiversity issues in the context of global change. Overall, however, further work along a broad range of taxa and populations is needed to confirm the generality of our findings.

## CONFLICT OF INTEREST

The authors declare no conflict of interests.

## AUTHOR CONTRIBUTIONS

**Anakena M Castillo:** Conceptualization (lead); Data curation (lead); Formal analysis (lead); Investigation (lead); Methodology (lead); Project administration (lead); Validation (lead); Visualization (lead); Writing‐original draft (equal); Writing‐review & editing (equal). **Luis De León:** Conceptualization (equal); Data curation (supporting); Formal analysis (supporting); Funding acquisition (lead); Investigation (supporting); Methodology (supporting); Project administration (lead); Validation (supporting); Visualization (supporting); Writing‐original draft (equal); Writing‐review & editing (equal).

### OPEN RESEARCH BADGES

This article has earned an Open Data Badge for making publicly available the digitally‐shareable data necessary to reproduce the reported results. The data is available at https://doi.org/10.5061/dryad.gf1vhhmp8.

## Supporting information

Fig S1‐S4

## Data Availability

COI sequences of *T. withei* are publicly available in Genbank (Accession Numbers: MW603763–MW603767). Experimental data associated with this study are available in the Dryad Digital Repository: https://doi.org/10.5061/dryad.gf1vhhmp8.
