# Supplementary material for: Evolutionary mismatch along salinity gradients in a Neotropical water strider
Source: Ecol Evol. 2021 Apr 9;11(10):5121–34. doi: 10.1002/ece3.7405 (PMC8131768; doi:10.1002/ece3.7405)
Supplement: Supplementary file 1 — Fig S1‐S4 [file ECE3-11-5121-s001.docx]

**Appendix**

**Fig. S1**. Fitness trade-offs for females (A) and males (B) along salinity a gradient in the water strider *Telmatometra withei*. Error bars showed mean and standard error.

**Fig. S2**. Variation in egg size (mm ^3^) in the water strider *Telmatometra withei* from fresh (FW) and brackish (BW) water environments.

**Fig. S3**. Number of egg (F1, F2) from brackish water population (panel A), and freshwater population (panel B). Number of immatures from brackish water population (panel C), and freshwater population (panel D). Number of adults from brackish water population (panel E), and freshwater population (panel F). Error bars showed mean and standard error per day. The x-axes showed day after immature emergence, as well as day of experiment until 90 days.

**Fig. S4**. Kaplan-Meier survival curves for adults of *Telmatometra withei* across salinity treatments. The data represent the probability of survival in freshwater (A) and brackish (B) populations during the 30 days of the common garden experiments. The p value represents statistically significance at 0.05 level.
